# Supplementary material for: AI-ming backwards: Vanishing archaeological landscapes in Mesopotamia and automatic detection of sites on CORONA imagery
Source: PLoS One. 2025 Aug 18;20(8):e0330419. doi: 10.1371/journal.pone.0330419 (PMC12360548; doi:10.1371/journal.pone.0330419)
Supplement: S1 Appendix — (DOCX) [file pone.0330419.s001.docx]

**S1 Appendix. Geographical coordinates**

All the coordinates are indicated using EPSG:3857 as reference system.

●  **GHR.079** (point): 4910150.9, 3931922.3

●  **GHR.078** (point): 4908749.1, 3931834.3

●  **GHR.077** (point): 4914790.3, 3947526.8

●  **GHR.036** (point): 4901124.7, 3928706.7
