# Supplementary material for: AI-ming backwards: Vanishing archaeological landscapes in Mesopotamia and automatic detection of sites on CORONA imagery
Source: PLoS One. 2025 Aug 18;20(8):e0330419. doi: 10.1371/journal.pone.0330419 (PMC12360548; doi:10.1371/journal.pone.0330419)
Supplement: Attachment — Submitted filename: PONE-reviewer-letter-2.0.pdf [file pone.0330419.s003.pdf]

ALMA MATER STUDIORUM
UNIVERSITÀ DI BOLOGNA

Dear Editor,

as suggested to us, we have revised our paper: "AI-ming backwards: Vanishing archaeological landscapes in Mesopotamia and automatic detection of sites on CORONA imagery", submitted for a possible publication to PLOS ONE.

We have taken into serious consideration ALL the comments and suggestions that both the Reviewers have made, and we have modified our paper accordingly (further adding one new Figure and one new Table). A letter answering to the comments of the two Reviewers is reported below.

It describes how we have modified our paper in response to all their comments. All the modifications (besides very minor changes elsewhere) we have made, have been highlighted with the green color in the text of the revised manuscript. In addition, we have also corrected both the Financial Disclosure and Competing Interests Statements, as per the Editor's indications.

Sincerely,

The Authors

Answers to Reviewers' Comments:

Reviewer 1

This is a paper focused on using deep learning techniques to auto-discover archaeological sites in Mesopotamia. The paper reflects a growing trend in the use of AI- based techniques to automate discovery of archaeological sites. *Overall, the paper is interesting* but requires more work prior to publication. I list some of my comments below

We thank the Reviewer for the kind words and the time and effort s/he put into the process of reading and evaluating our paper.

1- The review of previous literature misses quite a lot of developments in the area of remote sensing and archaeology using AI-based techniques. There have been various studies on also using mounds on maps or imagery to find sites. Although many of these do not focus on CORONA, perhaps incorporating these could be useful as it shows where the methodology is best understood in this growing literature. I think incorporating what has been done with UAVs, non-optical data, and other areas could be useful for better understanding many of these related techniques and ultimately how this work is different.

Our focus lay mainly in developing a new Deep Learning method (tested on a specific area) for utilizing historical sources in landscape studies, while it will fall to further studies to incorporate this method into the detailed assessment of the significance of settlement patterns and/or to compare different kinds of survey data acquisition (for example the elevation data of sites etc.). In this sense we would be worried to extend along the direction suggested by the Reviewer because we will lose focus on the real issue at the canter of our study. We hope the Reviewer will understand this choice.

2- The methodology, which is described and presented, needs a lot more description in the main text in my opinion. I think a better discussion on the steps and algorithm is critical. I understand that the details are best presented elsewhere, such as the code, but the paper should also have enough detail to give the reader they understand the mechanics of the technique. To me this needs more work and detail. There is not much discussion on the loss function, parameterization, and other steps

We concur with the whole picture depicted by the Reviewer on this specific point.

We think also that s/he should give more credit to the fact that there are precise limits to what we can/cannot repeat with respect to contents and ideas we have already published in previous papers. Not only that but we need to use a certain degree of conciseness to keep the current paper below some space limits.

In the end, since the Reviewer's comment is legitimate, we have decided to extend the Methods Section with a new Figure (Fig 2) and a one page and a half long explanation on how our deep convolutional network works to process high resolution archaeological image (lines 185-235).

In addition, we also felt sensible to the expectations of the Reviewer on the issues relative to the discussion on the performances of our neural network. Consequently, to give a more exhaustive answer to his/her requests, we have extended also the Results Section with a new Table (Table 1) and a one page long discussion concerning the accuracy achieved during the validation phase of our training activity (lines 286-307).

However, we remind here again that the validation phase is an integral part of the re-training activities. Hence, the corresponding results do not represent the ultimate measurement of how well our models recognize tells from new images. Nonetheless, these results can be interesting as they have given to us a preliminary assurance that our models have learnt effectively during re-training. Obviously, the fact remains that the results we obtained during the testing phase, with 20 new images that our neural network had never seen before, are more central to this paper and constitute a brand new result (along with the new discoveries).

At this point, we hope the Reviewer agrees that we have tried to better explain the point, extending the paper, while retaining conciseness.

3- Its good to see the tests done regarding accuracy. However, do we get a sense of sites being missed by survey and this technique? I know it is hard to tell without knowing what sites exist but random survey of the region studied could help identify what is being missed by these more focused approaches that are looking at areas seen on the results

Thank you for your observation, which gives us the opportunity to further clarify our approach. Indeed, the point you raise has been fully addressed in the study: during the two field campaigns conducted in 2023 and 2024, all potential sites identified through remote sensing—both those detected by traditional techniques and those predicted by deep learning models—were systematically investigated on the ground.

This comprehensive validation allowed us to empirically confirm the presence or absence of archaeological sites, thus providing a solid empirical basis for evaluating the efficacy and completeness of the adopted methodology. The in situ investigations, aimed at verifying potential archaeological sites identified through remote sensing, were conducted with the goal of rigorously validating the delineation of their boundaries, including exploration of adjacent areas.

This approach made it possible to refine the site perimeters established during the remote sensing phases and, where necessary, to redefine them based on field observations. The detailed results presented in the paper underline precisely the value of this integrated approach in identifying archaeological sites that might otherwise remain undetected by more traditional or spatially restricted techniques, as reflected in the revised paper in (lines 104-137).

4- Although tell sites are obviously common, many sites are flat in Mesopotamia or show little mounding. I understand that these might be difficult on optical data but this may mean other data should be used such as LiDAR (e.g., from UAVs), multispectral, and even higher resolution optical. It seems to me many sites could be missed with this technique. In fact, if one were to use Digital Elevation Data and use that to find sites, as tells become obvious with that data, could we not simply use that to find mounded sites? I would have tested how accurate this technique is relative to someone using DEM data to find sites and see which is more accurate. In any case, I think more discussion on the limitations of the research and why other data sources could not be used to find non-tell (mounded) sites is warranted as there are now a number of papers on finding mounded sites using deep learning in archaeology (see my comment on the literature review)

Thank you for your observation, which allows us to further clarify several relevant methodological aspects of our study. Firstly, it is important to specify that archaeological sites in the Mesopotamian floodplain appear almost exclusively as tells, artificial mounds characterized by varying elevations. Although some sites may display limited prominence, the presence of these mounds remains the predominant, though not the sole one, archaeological evidence in the region.

Regarding the suggestion to employ other data sources such as LiDAR, multispectral imagery, or ultra-high-resolution optical data, it must be emphasized that the choice of CORONA imagery is deliberate and methodologically sound. This choice precisely addresses the necessity of reconstructing an archaeological landscape predating the extensive anthropogenic impacts that have occurred in recent decades. Indeed, agricultural activities, modern canal construction, and infrastructure development have significantly damaged and altered archaeological sites, as thoroughly documented in our study. Specifically, our analysis has demonstrated that 38% of the identified sites have been entirely destroyed (31 sites of Fig 10), an additional 23% have lost more than half of their original area (19 sites of Fig10), and only 38% retain more than half of their initial extent (31 of Fig 10). These findings result from a meticulous comparison between the landscape depicted in the CORONA imagery from the 1960s and the current landscape visible in contemporary satellite imagery.

Furthermore, the detailed analysis of specific damage types clearly highlights that agricultural practices are responsible for the majority of site alterations, affecting 91% of the studied sites. This is followed by damage due to modern canal construction (42%) and recent building structures (39.5%). These alterations, ranging from minor modifications to complete destruction, have been rigorously documented and validated through both remote sensing and subsequent field surveys conducted in 2023 and 2024. (Note that not to make too complex this present study, we have deliberately made the decision not to provide all these numerical details on the causes behind destruction in the present manuscript).

Regarding the suggestion to employ other data sources such as UAV (drones), LiDAR, multispectral imagery, or ultra-high-resolution optical data, it is essential to clarify why these data sources were not considered in this study. These reasons can be grouped into three main categories.

First, each of these technologies has unique characteristics that make them particularly useful in specific and delimited contexts. UAVs, for instance, are generally employed in restricted and clearly defined areas, rendering their use logistically and economically unsustainable for a territory as extensive as approximately the 570 km² covered in this study. LiDAR technology, extensively utilized in areas characterized by dense vegetation cover, such as Mesoamerica and Cambodia, is crucial where penetration through thick vegetation is necessary; besides a similar objection may be raised as per the case above in terms of time and cost constraints for large areas (not to speak of getting the permits from competent authorities). Digital Elevation Models (DEM) are most effective for broader morphological analysis but are less precise in identifying heavily compromised archaeological sites.

Second, in the specific context of the Mesopotamian floodplain, many archaeological sites have suffered irreversible loss due to significant anthropogenic impacts in recent decades. Under these conditions, only historical imagery such as CORONA imagery from the 1960s can realistically reconstruct an archaeological landscape preceding recent intensive anthropization.

Third, a fundamental reason is the ethical and methodological choice of exclusively using open-source data. This ensures transparency, accessibility, and replicability of research—critical aspects of international scientific collaboration and archaeological data sharing.

Given the space limitations of the journal, we have not reported all this detailed explanation in our revised manuscript, but only a succinct summary available as a whole in (lines 104-137).

5- More discussion on usability of this would be good. For instance, on scaling the approach, time it takes to do this work, and benefits of this technique versus other techniques in the literature, such as Mask R-CNN, Random Forest, and other DL techniques

This comment is intriguing and well founded. As such, it requires a precise answer, divided over two different parts.

As to the first part of the question, the answer is simple and in line with the most part of similar DL systems used to process images of archaeological interest. On the system side, in fact, there are no particular scalability problems, in the sense that our convolutional neural network takes in the order of a few seconds to process a batch comprising some dozens of images. Nonetheless, being our system based on a human-in-the-loop approach, it is not possible to calculate the precise amount of time needed to human experts to conduct their evaluation on the system outputs (that is segmentation maps) and, in turn, to produce a refined dataset.

In conclusion, given these premises, we have decided it was no interesting to extend the paper to include a long discussion on this issue. We have simply added the information on the number of image processed per second at line X.

As to the second part of the question, it intercepts a similar comment provided by Reviewer 2 (Comment B, below), deserving the following answer.

Since the time we began our research activity in the field of Deep Learning applications to archaeology (which dates back to 2018) many other similar initiatives have taken shape and have been brought to the forefront of scientific discussion.

We are well aware that studies, like those conducted by Menze et al 2006, Soroush et al 2020, Guyot et al 2018, Orengo et al 2020, and many others, have made relevant advancements to this field. Our attention to these results is **intense** and witnessed by the fact that both the works by Guyot et al and Orengo et al were **already cited** both in our previous works, for example [14], and in the **current paper** as well (**!**), together with some other relevant researches.

Nonetheless, this point needs a deeper treatment to provide a precise motivation why not any study in this field always deserves a citation with each new paper proposed.

There are (at least) two axes along which experts can look at similarities or differences in all these researches. The first one is concerned with the remote sensing methodologies employed to gather the data on which AI techniques will work. Using passive or active approaches, satellite or aircrafts, radar or LiDAR or any other typology of sensors emitting their own signals, or a combination of all of them, constitutes a relevant difference that extends till the point of a distinction (or divergence) in the meaning of the results that are obtained. Not to consider the issue of the type of data, as working with new rather than old (e.g., CORONA) high resolution images represents another important source of differentiation.

The second axis is that of the distinction between ML and DL. Machine Learning (ML) are algorithms that learn from **structured** data to predict outputs and discover patterns in that data. Deep Learning (DL), instead, is always based on highly complex neural networks that mimic the way a human brain works to detect patterns in large **unstructured** data (like images). A traditional ML algorithm can be something as simple as linear regression or a search in a decisional tree (e.g., Random Forest), the driving force behind being often ordinary statistics. DL algorithms, instead, should be regarded as a sophisticated and mathematically complex evolution of ML. To achieve this result, DL approaches use a layered structure of algorithms, called an artificial neural network, with a specific design based on a cascade of several different computational blocks, inspired by the biological neural network of the human brain, and leading to a process of learning that is far more capable than that of standard ML models. Nonetheless, with DL we fall often into an excess of inference, to which we cannot always give a formal explanation. An extension of this discussion, tailored to the archaeological field, is reported in Gattiglia, 2025, and it is now cited in our paper. Consequently, applying ML or DL makes another important difference, being often unfair or even nonsensical, in the light of the explanation above, a comparison between studies that adopt different approaches.

In the end, given all the premises above, we hope that both the Reviewers can look at (the comprehensive context of) our study as an *unicum*: (i) learning with ANN + attention mechanisms, ii) using transfer learning to combine the knowledge provided by both new and old (CORONA) satellite images, and iii) the tells of the Mesopotamia floodplain as single subject of investigation. Along this line of sense, we are not sure that the quality of our paper would increase simply by including one-to-one comparisons with other specific papers that have followed different approaches, rather we feel we are doing our duty of responsible researchers by witnessing the level of productivity of the entire community in this field by citing some selected studies.

In conclusion, we recognize that the comments provided by both Reviewers 1 and 2 are legitimate and deserve an answer, and to this aim we have extended our paper (Introduction Section) with all the references suggested by Reviewer 2, plus some others (References 11-15), all together with a summary of the discussion above (lines 52-81). Reviewers should finally consider that the References we have cited amount now to **48 papers**.

Reviewer 2

A- Overall, this is an interesting article employing a methodology which I hope to become more developed and common in broadscale settlement analysis.

We thank the Reviewer for the kind words and the time and effort s/he put into the process of reading and assessing our paper.

B- However, a concern is that the authors present this method as a novel approach, when uses of machine learning algorithms have been employed to this end before in similar areas (Menze et al 2006, Soroush et al 2020, Guyot et al 2018, Orengo et al 2020, etc.). It is concerning that other studies such as these are not cited.

This legitimate criticism aligns well with a similar comment provided by Reviewer 1 (Second part of Comment 5, above), and it deserves convincing answers which follow, here, again.

Since the time we began our research activity in the field of Deep Learning applications to archaeology (which dates back to 2018) many other similar initiatives have taken shape and have been brought to the forefront of scientific discussion.

We are well aware that studies, like those conducted by Menze et al 2006, Soroush et al 2020, Guyot et al 2018, Orengo et al 2020, and many others, have made relevant advancements to this field. Our attention to these results is **intense** and witnessed by the fact that both the works by Guyot et al and Orengo et al were **already cited** both in our previous works, for example [14], and in the **current paper** as well (**!**), together with some other relevant researches.

Nonetheless, this point needs a deeper treatment to provide a precise motivation why not any study in this field always deserves a citation with each new paper proposed.

There are (at least) two axes along which experts can look at similarities or differences in all these researches. The first one is concerned with the remote sensing methodologies employed to gather the data on which AI techniques will work. Using passive or active approaches, satellite or aircrafts, radar or LiDAR or any other typology of sensors emitting their own signals, or a combination of all of them, constitutes a relevant difference that extends till the point of a distinction (or divergence) in the meaning of the results that are obtained. Not to consider the issue of the type of data, as working with new rather than old (e.g., CORONA) high resolution images represents another important source of differentiation.

The second axis is that of the distinction between ML and DL. Machine Learning (ML) are algorithms that learn from **structured** data to predict outputs and discover patterns in that data. Deep Learning (DL), instead, is always based on

highly complex neural networks that mimic the way a human brain works to detect patterns in large **unstructured** data (like images). A traditional ML algorithm can be something as simple as linear regression or a search in a decisional tree (e.g., Random Forest), the driving force behind being often ordinary statistics. DL algorithms, instead, should be regarded as a sophisticated and mathematically complex evolution of ML. To achieve this result, DL approaches use a layered structure of algorithms, called an artificial neural network, with a specific design based on a cascade of several different computational blocks, inspired by the biological neural network of the human brain, and leading to a process of learning that is far more capable than that of standard ML models. Nonetheless, with DL we fall often into an excess of inference, to which we cannot always give a formal explanation. An extension of this discussion, tailored to the archaeological field, is reported in Gattiglia, 2025, and it is now cited in our paper. Consequently, applying ML or DL makes another important difference, being often unfair or even nonsensical, in the light of the explanation above, a comparison between studies that adopt different approaches.

In the end, given all the premises above, we hope that both the Reviewers can look at (the comprehensive context of) our study as an *unicum*: (i) learning with ANN + attention mechanisms, ii) using transfer learning to combine the knowledge provided by both new and old (CORONA) satellite images, and iii) the tells of the Mesopotamia floodplain as single subject of investigation. Along this line of sense, we are not sure that the quality of our paper would increase simply by including one-to-one comparisons with other specific papers that have followed different approaches, rather we feel we are doing our duty of responsible researchers by witnessing the level of productivity of the entire community in this field by citing some selected studies.

In conclusion, we recognize that the comments provided by both Reviewers 1 and 2 are legitimate and deserve an answer, and to this aim we have extended our paper (Introduction Section) with all the references suggested by Reviewer 2, plus some others (References 11-15), all together with a summary of the discussion above (lines 52-81). Reviewers should finally consider that the References we have cited amount now to **48 papers**.

C- I am also concerned at the apparent conflation of "tells" with archaeological sites more broadly. One of the main benefits of this technology is that it should not just be able to identify tells, which are easily identifiable without ML. However, throughout the article, it is difficult to understand if the authors are referring only to the identification of tells (which seems to be the case) or all archaeological sites. Certainly, there are plenty of "flat" sites

Thank you for raising this point, which provides an opportunity to further clarify the scope and limitations of the methodology presented.

In the context of this study, the term "archaeological site" has been used primarily in reference to tell-type settlements, which represent the most common and morphologically distinct form of occupation in the alluvial plain of southern Mesopotamia. As described in the text, tells are artificial mounds formed by the stratified accumulation of materials resulting from long-term human occupation, and they are particularly well-suited to detection through both traditional remote sensing techniques and automated approaches, due to their topographic prominence and characteristic shape.

The decision to focus on tells does not stem from any intrinsic limitation of the technology, but rather from a deliberate methodological choice aligned with the specific objectives of this phase of the project, which aimed to evaluate the effectiveness of AI models in detecting clearly defined settlement mounds using historical satellite imagery (CORONA). This choice was also driven by the need to test the model on well-documented features whose archaeological nature could be confidently confirmed through field validation.

We are fully aware, however, of the existence and relevance of low-relief or flat archaeological sites, such as seasonal campsites, cemeteries, or temporary occupations, which do not necessarily appear as raised features. It is important to emphasize, however, that such site types are relatively rare in the southern Mesopotamian alluvial plain, especially when compared to the predominance of tells. Precisely because of their subtle morphology, these sites present different challenges for detection and, indeed, were not included in the model's training dataset or within the validation framework adopted in this study.

We are in the end confident that this long explanation can be sufficient to convince the Reviewer. As to the change to the revised manuscript in response to this answer, we did not identify a particular portion where all this is discussed, as this pervades all the manuscript in general.

D- My second main concern with this article is that the AI/ML approach used to identify sites does not actually assist the preexisting survey methods in a meaningful way. If I am reading this correctly, then it appears that the ultimate result of this study is 4 sites and 4 false positives identified that were not initially discovered during survey. However, when compared to the 80+ sites that were identified and surveyed, this number seems almost negligible and not at all like a "real breakthrough" that the authors purport it to be. In fact, in the conclusions section they suggest that these "discovered" sites by the model had "the appropriate morphological characteristics" - does that mean that normal exploratory foot survey would have discovered these sites? Ultimately, it doesn't seem that the model made much of a difference at all to the results of a survey that was using CORONA imagery to identify potential sites in the first place. The authors have given information for the accuracy of the different models they tested, but how does it compare to the accuracy of "normal" site identification with satellite imagery? While the accuracy here is 50/50 in terms of "newly identified" sites, in my experience a normal positive-false positive ratio is more akin to 60-75%-40-25%. (It is also unclear why CORONA makes such a difference in the case of this model - I'm assuming it's the result of modern urbanization and industrial agriculture which have now obscured these sites, but this is unclear in the text itself)

We appreciate the reviewer's attention and constructive feedback. We wish to clarify several fundamental aspects of our approach by highlighting the integrated nature of our process and delineating the sequential phases that culminated in the presented results, for the benefit of the Reviewer.

1. Distinct yet Complementary Phases of the Process

It is important to emphasize that the work undertaken by the archaeologists and the training of the Deep Learning model represent two separate phases, each with its own objectives and methodologies, which were subsequently integrated in a synergistic process. Initially, the model underwent a training, validation, and testing phase – the latter evaluated through quantitative metrics (refer to Tables 3 and 4 respectively at a pixel level and tells as a whole). Notably, the model achieved significant performance metrics, with an accuracy of 0.90 and a recall of 0.88 on 20 unseen images. These results should be considered when evaluating the model alone.

2. Qualitative Comparison between Models

To underscore the contribution of the CORONA imagery, we conducted a qualitative comparison between the "bing/corona" model and the conventional "bing" model, which relies on standard satellite imagery for site identification. This comparison demonstrated that the inclusion of the additional contextual information provided by CORONA enables a more effective detection process; specifically, the "bing/corona" model correctly identified three additional instances which the "bing" model erroneously classified as false negative, as extensively discussed in the paper.

3. Heatmap Generation and Site Selection

The results highlighted (namely, four confirmed sites and four false positives) do not represent the direct output of the model. Rather, they are the product of a comprehensive and multi-step procedure. Initially, the model generated a heatmap containing all predictions within the area of interest (as illustrated in Fig. 3). Subsequently, leveraging archaeological knowledge and techniques, these predictions were compared with the findings from traditional remote sensing analyses. Predictions corresponding to areas already verified, either positively or negatively, are discarded. From the remaining set, only zones of particular archaeological interest, selected based on their morphological characteristics and their location in areas not previously considered of attention, are taken into consideration. These zones are then subjected to field verification, which ultimately can either confirm the sites (or not). It is important to note that the field verification phase functions as an additional test enabled by the integration of the model into the archaeological research process. This is exactly what happened and is discussed in our paper with the four new discoveries.

4. Methodological Innovation and Integrated Contribution

Our approach transcends the mere application of remote sensing techniques. By integrating the predictive capability of the AI/ML model with the specialized knowledge of archaeologists, the methodology generates an added value that is not immediately quantifiable solely in numerical terms. In essence, the innovative contribution lies in the integrated methodology, which facilitates the identification of potential sites in a more targeted and efficient manner, even in contexts where traditional methods may prove less effective. In this sense, the reported results (four true positives and four false positives) should not be interpreted as the sole output of the model nor as the primary result to be presented. Instead, they represent the convergence point of a multi-phase process (human in the loop) in which each component – the AI/ML model and the archaeological knowledge – plays a critical role in enhancing the overall quality of site detection. After all, our article reports on a new method which has been tuned so that it may be now more extensively tested and adopted by the scientific community.

Finally, given the extension of this newly added explanation, we do not refer here to some specific changed portion of the paper in particular, but to the whole revised manuscript in general.

E- I think that an article discussing this approach has merit, but should be tested on a larger dataset with more distinct results to merit publication in this journal. My conclusion is to reject this manuscript, but with hope that the authors will continue to develop and apply this model to further material to produce meaningful results in the future

We thank the reviewer for giving us the opportunity to further clarify this point. **The most comprehensive dataset currently at our disposal for the study of the archaeological landscape of the Mesopotamian alluvial plain consists of the complete corpus of known archaeological sites, developed within the framework of the Floodplains project, the results of which have already been published**. The preliminary analyses we conducted on this dataset were aimed at building a flexible model, designed to be easily adaptable to different operational scenarios, and its effectiveness has already been verified in that context. The challenge addressed in this new study was therefore to test the model in a real-world application, namely the survey of a previously unexplored region, in order to assess its actual potential within a fieldwork setting. Clarified this situation, we are confident that the Reviewer can agree with us that the paper does not need any further change.

.
