# Supplementary material for: AI-ming backwards: Vanishing archaeological landscapes in Mesopotamia and automatic detection of sites on CORONA imagery
Source: PLoS One. 2025 Aug 18;20(8):e0330419. doi: 10.1371/journal.pone.0330419 (PMC12360548; doi:10.1371/journal.pone.0330419)
Supplement: Attachment — Submitted filename: Ultimate-Letter-to-Reviewer.pdf [file pone.0330419.s004.pdf]

Dear Editor,

as suggested to us, we have revised our paper: “AI-ming backwards: Vanishing archaeological landscapes in Mesopotamia and automatic detection of sites on CORONA imagery”, submitted for a possible publication to PLOS ONE.

We have taken into serious consideration ALL the comments and suggestions that the Reviewer has made, and we have modified our paper accordingly (further adding one reference and one new texte). A letter answering to the comments of the Reviewer is reported below.

It describes how we have modified or not our paper in response to all his comments. The modifications we have made, have been highlighted with the reddish color in the text of the revised manuscript.

Sincerely,
The Authors

Answers to the Reviewer’s Comments:

1) The review of existing literature in this area is still somewhat minimal. I know this is not a review paper, so referencing all works is not that critical but I think more around the specific architecture chosen and the background into that would help because there are other architectures one could have chose and it is not clear to me why this particular method say is better or more appropriate over other CNN-based or DL models/architectures

Answer to 1)

We feel confident in asserting that a list of 48 references, with more than half addressing issues related to AI, allied technologies, and their application in the archaeological domain, is already sufficiently broad and encompassing as to preclude the need for further expansion.

Nonetheless, solely to provide a further confirmation regarding the type of impact our work on the use of AI techniques on satellite data has had in the scientific world, even outside the archaeological context, we are adding a very recent reference, number 49 below.

In this reference, experts from the Chinese government, from one of the most prestigious Chinese laboratories (located in Wuhan), **engage with our method**, aiming

to use satellite data for calculating plausible routes for orbiting towards the planet Jupiter.

[49] Afzal Z, Yan J, Barriot J-P, Sun S, Haider Z, et al. Evaluating the contribution of Tianwen-4 mission to Jupiter's gravity field estimation using inter-satellite tracking. Astronomy & AstroPhysics, 2025, doi: 10.1051/0004-6361/202554439

In closing this issue, concerning a potential additional comparison with other neural networks, it seems clear that the Reviewer fails to adequately acknowledge that there is not a single definitive number of neural network types, as the field is constantly evolving with new architectures and variations being developed. For the record, here are the main categories:

- Feedforward Neural Networks (FFNNs):
- Perceptron (Single-Layer Perceptron): The simplest form, with only one layer of neurons.
- Multi-Layer Perceptron (MLP): The most common and fundamental type, with at least one hidden layer. It forms the basis for many other architectures.
- Recurrent Neural Networks (RNNs): Designed to handle sequential data, where the output at one step depends on the input and the hidden state of the previous step.
- Long Short-Term Memory (LSTM): An RNN variant that solves the vanishing gradient problem, excellent for long sequences.
- Gated Recurrent Unit (GRU): Similar to LSTMs but simpler, with fewer parameters.
- Bidirectional RNN (Bi-RNN): Processes sequences forward and backward to capture context from both directions.
- **Convolutional Neural Networks** (CNNs): Excellent for analyzing data with a grid-like topology, such as images (2D) or time series (1D). For example: LeNet, AlexNet, VGG, ResNet, Inception, MobileNet are all xamples of specific CNN architectures that have become standard for image recognition (just our case. Also: 1D, 2D, 3D CNNs: Depending on the dimensionality of the input data.
- Generative Adversarial Networks (GANs): Composed of two competing networks (a generator and a discriminator) that work together to produce new, realistic data.
- DCGAN (Deep Convolutional GAN), with StyleGAN, BigGAN as more advanced examples.
- Autoencoders: Networks that learn a compressed representation (encoding) of the input data and then attempt to reconstruct the original input (decoding). Used for dimensionality reduction and denoising. Example: Vanilla Autoencoder
- Variational Autoencoder (VAE): Used for data generation.
- Denoising Autoencoder, Sparse Autoencoder: Variants for specific purposes.

- Transformer Networks: Revolutionary for Natural Language Processing (NLP), they rely on a self-attention mechanism to weigh the importance of different parts of the input. For example, BERT, GPT (Generative Pre-trained Transformer) are well-known examples of Transformer-based models.
- Radial Basis Function Networks (RBFNs): Used for function approximation and classification, with neurons in the hidden layer that have radial basis activation functions.
- Self-Organizing Maps (SOMs): A type of unsupervised neural network used for dimensionality reduction and visualizing high-dimensional datasets.
- Liquid State Machines (LSMs) / Echo State Networks (ESNs): A type of recurrent neural network that uses a "reservoir" of neurons with fixed, random connections, where only the output layers are trained.
- Neural Turing Machines (NTMs) / Differentiable Neural Computers (DNCs): Combine neural networks with external, addressable memory, allowing them to learn complex algorithms.

And the list list **only provides an overview** of the main categories, and some of the most prominent architectures. Each category, in turn, includes **countless variations** and specializations.

What is important is that **the choice of a neural network type** largely **depends** on the **specific data to be processed** and obviously the problem to be solved. **With satellite images,** like in our case, the use odìf a **DCNN** of **MaNet/Imagenet -type** is a **standard practice** and**, among experts**, **should not necessitate additional clarification** beyond that already given.

2) Validation test are somewhat limited. A broader range of statistical tests could be applied to test how accurate and reliable the quality of the image identification is. Usually I would expect a wider range, including RMSE and other tests to be there

Answer to 2)

We honestly find it very **challenging to believe** that requesting RMSE test values is appropriate in this context.

We are sure that the Reviewer knows that **Root Mean Squared Error** is a common metric typically used in **regression problems** (which **is not exactly our case**) to quantify the average magnitude of the errors between predicted values and actual observed values.

In our specific situation (considering that the subject are **images**), any expert in the scientific world would agree that the measures we have provided - **IoU**, **bIoU**, **MCC**, and

then **Accuracy** and **Recall** - are far **more significant** and **impactful** for **validating the results** we have obtained than RMSE. Hence**, the decision not to measure RMSE**, which **would border** on the **nonsensical**, statistically speaking.

3) I still think more can be done with the non-tell based sites but I can accept to leave that for now. Some discussion at least as to how this could be an improvement in the future might be warranted (i.e., how to improve non-tell based site identification as this would really be transformative if we can go beyound mound identification).

Answer to 3)

We have inserted the following text:

While our work has focused on tell-based sites—characterized by distinctive morphologies well-suited to automatic segmentation—it is worth noting that extending this approach to non-mounded contexts would represent a theoretically significant development. However, the lack of recurrent morphological features, the semantic heterogeneity of archaeological traces, and the current scarcity of annotated datasets make such a direction presently difficult to pursue. Advancing in this area would require fundamentally different classification strategies, substantial refinement of source data, and a methodological rethinking that goes beyond the aims and operational scope of the present study.
